# Characteristics and Outcomes of Failure to Thrive in Pediatric Patients Referred to the King Abdulaziz Medical City Pediatric Clinic

**DOI:** 10.7759/cureus.55491

**Published:** 2024-03-04

**Authors:** Mesbah Jari Alshumrani, Abdulaziz M Gammash, Basil A Alzahrani, Orjwan S Badghaish, Lama Alajlani, Atheer A Alzahrani, Albatool Ali, Mohamed E Ahmed

**Affiliations:** 1 Department of Paediatrics, King Saud Bin Abdulaziz University for Health Sciences, Jeddah, SAU; 2 College of Medicine, King Saud Bin Abdulaziz University for Health Sciences, Jeddah, SAU; 3 College of Sciences & Health Professions, King Saud Bin Abdulaziz University for Health Sciences, Jeddah, SAU

**Keywords:** nutrition, failure-to-thrive, child nutrition, malnutrition, malabsorption

## Abstract

Background: Failure to thrive (FTT) in pediatric populations is a diagnostic challenge with implications for growth and development. Despite its prevalence, detailed epidemiological data, especially concerning organic versus non-organic etiologies, are sparse. This study examines the prevalence, characteristics, and outcomes of organic and non-organic FTT in a pediatric outpatient setting at King Abdulaziz Medical City, Jeddah.

Methods: This retrospective chart review included pediatric patients aged three months to 14 years diagnosed with FTT at KAMC from 2016 to 2023. FTT was defined by weights below the 3rd percentile or a decline across two major growth percentiles. Patients were stratified into organic and non-organic FTT groups based on predefined criteria. Prevalence rates, clinical characteristics, and outcomes were compared to draw distinctions between the two categories.

Results: Out of 349 evaluated patients, organic FTT was present in 160 patients (45.8%), with gastrointestinal conditions and endocrine disorders being the most common etiologies. Non-organic FTT, accounting for 189 (54.2%) of cases, was primarily associated with inadequate nutritional intake and behavioral factors. Notably, the non-organic FTT group exhibited a significantly higher rate of condition resolution (45.0%) compared to their organic counterparts (32.5%). Furthermore, significant laboratory parameter differences were noted, indicating a higher white blood cell count in organic cases, among other findings.

Conclusions: Non-organic FTT was more prevalent and demonstrated higher resolution rates, suggesting better outcomes with timely intervention and appropriate care strategies. The study advocates for increased educational efforts for caregivers and healthcare providers and calls for further research to explore effective management protocols for FTT.

## Introduction

Failure to thrive (FTT) is a term used to describe inadequate growth or the inability to maintain growth, usually in early childhood, due to insufficient caloric intake [1]. The diagnostic bases of FTT include weight for length below the 5th percentile, body mass index (BMI) below the 5th percentile, calculated as weight in kilograms divided by the square of height in meters for age, or a sustained drop in growth velocity, in which weight for age or weight for length/height decreases by two major percentiles (95th, 90th, 75th, 50th, 25th, 10th, and 5th percentiles) over time [2].

FTT has multiple etiologies. The potential causes of a child’s failure to gain weight can be classified into three broad categories: inadequate calorie intake, inadequate calorie absorption, and increased caloric demand. Diseases such as celiac disease, pancreatic insufficiency, congenital heart disease, and renal disease can lead to inadequate absorption and increased calorie expenditure. Examples of inadequate caloric intake due to non-organic causes include lack of food availability, neglect, breastfeeding difficulties, and improper formula preparation [1,3].

Another way to categorize FTT is through the organic and non-organic causes. Organic FTT refers to FTT that is secondary to a known disease. Meanwhile, non-organic FTT is caused by insufficient caloric intake without a known illness. It might be related to non-organic feeding disorders (NOFDs), which are negative eating behaviors defined by reduced food intake for no apparent organic reason, food rejection, selective food intake, and dread of feeding. In addition to NOFDs, micronutrient deficiencies have been linked to non-organic FTT. It is a condition where key nutrients such as folic acid, iron, vitamins, and magnesium are deficient due to familial, clinical, and socioeconomic issues. Even though these two categories are distinct, they might overlap. For example, a child might refuse to eat food due to an organic condition. The two categories can be distinguished by excluding organic conditions and relying on physician diagnosis to assess the underlying factors [4,5].

Although many children with FTT seem to have normal function early in the course of the disease, it remains an important area to recognize because it can result in developmental delay and other long-term complications in a developing child [6,7].

FTT appears to be a prevalent issue affecting children on a global scale; however, epidemiological studies regarding this problem are inadequate and out-of-date to address the burden of the disease, which can occur in approximately 10% of pediatric patients in primary care and approximately 5% of hospitalized children in the United States [8,9]. Franceschi et al. conducted a prospective single-center study and found that the prevalence of FTT was 2.6% (82 of 3127) in subjects hospitalized for five years [2]. A study in Turkey found that non-organic causes accounted for 78.6% of cases with mainly nutritional etiologies, which include deficiencies in minerals (magnesium, calcium, and phosphate), trace elements (iron and zinc), and vitamins (B12, A, D, E, and folic acid) [10]. However, another study conducted in Lebanon estimated that the most common etiology of FTT is organic, accounting for 77% of the cases [11]. Furthermore, the study also found that growth hormone deficiencies accounted for 23% of cases. Additionally, a study conducted in Italy found that organic causes accounted for 43 % of cases, with gastrointestinal and genetic causes being the most common [2].

Since there is a lack of new data regarding the outcomes of organic and non-organic FTT in international and local platforms, this study aims to be the first to report the characteristics and outcomes of organic and non-organic FTT in Jeddah.

## Materials and methods

Study design

This was a retrospective chart review study conducted on pediatric patients followed up in King Abdulaziz Medical City (KAMC), Jeddah, from the 1st of January 2016 to the 28th of August 2023. The main objective of this study was to report the characteristics and outcomes of organic and non-organic FTT in outpatients referred to the KAMC pediatric clinic. The study was approved by the Institutional Review Board (IRB) of King Abdullah International Medical Research Center (KAIMRC)(IRB/1069/23).

Identifications of study participants

The study included all pediatric patients aged three months to 14 years who were confirmed to have FTT based on growth parameters of weight below the 3rd percentile or weight that has decreased by two major percentiles from the baseline on the WHO chart for patients below two years of age and patients with weight below the 3rd percentile or weight that has decreased by two major percentiles from the baseline on the CDC chart for patients above two years of age. Patients with a BMI between the 5th and 85th percentile, patients hospitalized in the pediatric ward with a secondary diagnosis of FTT, and those with incomplete data were excluded as the study setting is the pediatric outpatient clinic. Based on the sample size calculation, considering a margin of error of 5% and a confidence interval of 95% and using the Raosoft sample size online calculator (Raosoft Inc., Seattle, USA) for retrospective studies, the required sample size was 249. The study encompassed a total population of 631 participants, out of which 349 satisfied the inclusion criteria established for this study. 

Data collection

Electronic medical records were used to collect the data, and laboratory parameters such as hemoglobin level, white blood count, C-reactive protein, platelets, thyroid stimulating hormone, ferritin, and vitamin D were compared between the two groups. Additionally, the patient's age at referral, gender, height, height after a year, weight, and weight after a year were recorded. The parameters were gathered in accordance with the hospital protocol, which mandates the administration of these tests to all patients suspected of suffering from FTT. To ensure the anonymity of the participants, their names and medical record numbers were not collected. 

Data analysis

The collected data were revised and prepared in Excel prior to data analysis. The data was collected from the period between the 1st of January 2016 to the 28th of August 2023. JMP statistical software (JMP, Cary, NC) was used to analyze the data. Descriptive statistics included the frequency and percentage of categorical variables and means, median, SD, and IQR were calculated for continuous variables. The chi-square test was performed to examine the association between categorical variables. Independent samples t-test and Mann-Whitney non-parametric tests were conducted to explore the significant differences between the means of laboratory parameters. Statistical significance was set at p < 0.05.

## Results

A total of 349 participants were included in the study, of which 160 participants (45.8%) were classified into the organic cause group and 189 into the non-organic cause group. Male children were 188 (53.9%), and the mean age at referral was 5.2 years with 3.0 SD. The mean height of patients included in the study was 99.5 cm with 21.2 SD, and the weight mean was 13.7 kg with 5.5 SD. The mean ages at referral among the organic group were 4.7 years and 5.6 years among the non-organic groups, respectively (Table 1).

**Table 1:** Basic characteristics of participants

Variable	All (349)	Organic (160)	Non-organic (189)
Gender			
Female (n %)	161 (46.1)	74 (46.3)	87 (46.0)
Male (n %)	188 (53.9)	86 (53.7)	102 (54.0)
Age at referral (years) (mean ±SD)	5.2± 3.0	4.7± 3.1	5.6± 2.8
Height (mean ±SD)	99.5± 21.3	94.3± 22.5	103.9± 19.2
Weight (mean ±SD)	13.7± 5.5	12.4± 5.7	14.8± 5.0
Height after a year (mean ±SD)	105.± 18.7	101.5± 20.4	108.9± 16.5
Weight after a year (mean ±SD)	15.7± 5.7	14.8± 6.2	16.4± 5.1

Table 2 shows the differences in laboratory parameters between the organic and non-organic groups of children. A significant difference in hemoglobin (HGB) means was identified, with the non-organic group having a higher mean of 12.5 compared to the organic group of 11.8. The organic group had a significantly higher mean white blood cell count (WBC) (8.5) than the non-organic group (7.5). Ferritin and vitamin D levels were significantly higher in the organic group. A significant association was found between resolution and children’s groups (P < 0.05); 45.0% of non-organic cases were resolved compared with 32.5% of cases in the organic group. Participants were considered resolved when their weight and height were above the 5th percentile.

**Table 2:** Lab parameters ^1^Median (IQR), ^*^Significant difference, HGB: Hemoglobin, WBC: White blood count, CRP: C-reactive protein, PLT: Platelets, TSH: Thyroid-stimulating hormone.

Variable	All (349)	Organic (160)	Non-organic (189)	P-value	Unadjusted OR
HGB (g/dl) (mean ±SD)	12.2± 1.5	11.8± 1.8	12.5± 1.2	0.003*	
WBC (/L) (mean ±SD)	7.9± 3.1	8.5± 3.6	7.5± 2.5	0.002*	
CRP (mg/L)^1 ^ (n %)	1.3 (5.6)	2.6 (10.5)	1.0 (4.0)	0.141	
PLT (/L) (mean ±SD)	368± 120	372± 134.2	364± 107.3	0.557	
TSH (mIU/L) (mean ±SD)	2.5± 1.5	2.7± 1.7	2.4± 1.4	0.084	
Ferritin (ug/L)^1^ (n %)	24.0 (29.0)	24.6 (23.2)	24.0 (29.1)	0.047*	
Vitamin D (nmol/L) (mean ±SD)	50.4± 23.4	54.3± 27.8	47.8± 19.4	0.039*	
Resolved					
No (n %)	212 (60.7)	108 (67.7)	104 (55.0)	0.017*	1.72
Yes (n %)	137 (39.3)	52 (32.5)	85 (45.0)		

**Figure 1:**
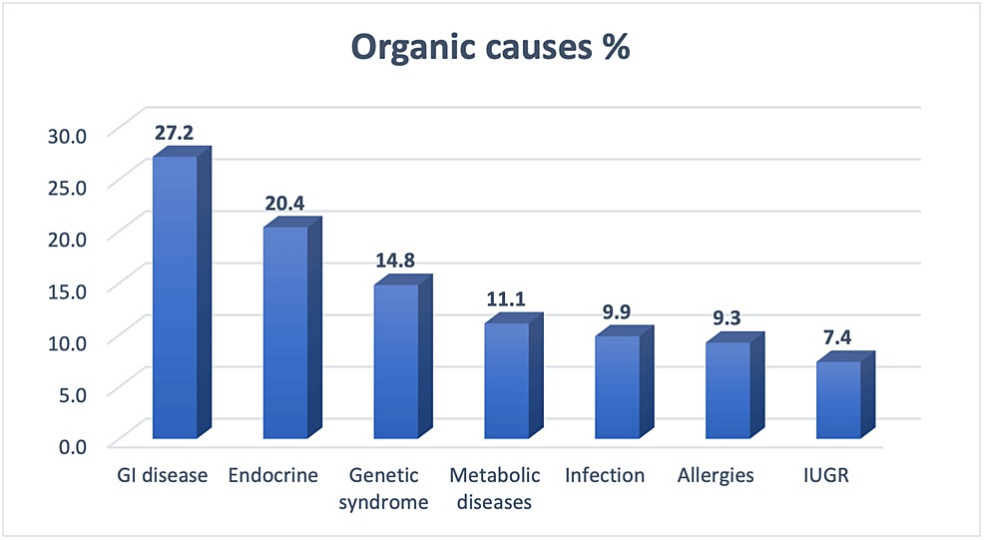
Failure to thrive organic causes. GI: Gastrointestinal; IUGR: Intrauterine growth retardation.

The distribution of FTT organic causes in Figure 1 reveals that gastrointestinal (GI) disease was the most common cause (27.2%), followed by endocrine disease (20.4%), and the lowest percentage was 7.4 for intrauterine growth retardation (IUGR).

A significant association was identified between organic FTT and FTT resolution (P < 0.05). Approximately 56% of the infection cases were resolved, followed by IUGR (50%), and metabolic disease had the lowest percentage of FTT resolution (11.1%) (Table 3).

**Table 3:** Organic causes by resolve. ^1^Median (IQR); ^*^Significant association; GI: Gastrointestinal; IUGR: Intrauterine growth retardation.

Cause	Resolved	Not resolved	P-value
GI disease (n %)	16 (36.4)	28 (63.6)	0.022*
Endocrine^1^ (n %)	7 (21.2)	26 (78.8)	
Infection (n %)	9 (56.3)	7 (43.7)	
Genetic syndrome (n %)	5 (20.8)	19 (79.2)	
IUGR (n %)	6 (50.0)	6 (50.0)	
Allergies^1 ^ (n %)	7 (46.7)	8 (53.3)	
Metabolic diseases (n %)	2 (11.1)	16 (88.9)	

The analysis of significant differences in laboratory parameters by FTT non-organic causes revealed significant differences (P < 0.05) in the thyroid-stimulating hormone (TSH) means, and the TSH level was higher in the inadequate intake group than in the behavioral difficulties group (Table 4).

**Table 4:** Lab parameters and resolve by non-organic causes. ^*^Significant difference; HGB: Hemoglobin, WBC: White blood count, CRP: C-reactive protein, PLT: Platelets, TSH: Thyroid-stimulating hormone. ^**^inadequate intake and behavioral feeding difficulties were included after clinical dietitian assessment

Variable	Inadequate intake** (125)	Behavioral** (65)	P-value
HGB (g/dl) (mean ±SD)	12.4± 1.2	12.5± 1.2	0.971
WBC (/L) (mean ±SD)	7.7± 2.6	7.1± 2.2	0.074
CRP (mg/L) (mean ±SD)	1.1 (4.1)	2.6 (10.1)	0.322
PLT (/L) (mean ±SD)	371.4± 118.0	355.3± 85.3	0.331
TSH (mIU/L) (mean ±SD)	2.6± 1.4	2.1± 1.1	0.036*
Ferritin (ug/L) (mean ±SD)	24.0 (30.0)	25.0 (22.1)	0.583
Vitamin D (nmol/L) (mean ±SD)	48.5± 20.1	46.5± 17.9	0.277
Resolve			
Yes (n %)	52 (41.6)	33 (50.8)	0.227
No (n %)	73 (48.4)	32 (49.2)	

Height and weight gain results are shown in Table 5. Among the organic groups, the highest percentage of height gain was found among the IUGR (13.5%) group, followed by the IUGR (10.2%) group among children with allergies.

**Table 5:** Height and weight gain after a year. IUGR: Intrauterine growth retardation.

Cause	Height mean difference	% of increase	Weight mean difference	% of increase
Organic				
GI disease	6.6	6.7	3.3	25.6
Endocrine	5.3	5.3	2.2	15.8
Infection	7.7	7.5	2.2	15.6
Genetic syndrome	7.8	9.2	1.6	15.5
IUGR	12.6	13.5	3.5	26.1
Allergies	9	10.2	2.6	23.2
Metabolic diseases	6.6	8.2	1.9	21.1
Non-organic				
Inadequate intake	5	4.8	1.6	10.8
Behavioral	4.8	4.6	1.7	11.4

## Discussion

FTT describes a pattern of impaired growth and development in children, rather than a single specific diagnosis. This is typically characterized by weight falling below the second percentile for a child's corrected gestational age and sex on a standard growth chart, paired with a discrepancy between weight and height gain rates. In clinical practice, FTT is recognized as a relatively common concern, affecting approximately 5-10% of children in primary care settings and 3-5% in referral settings, according to existing studies [12]. While insufficient caloric intake often plays a primary role, a multitude of medical and psychological factors can contribute to FTT [13].

This study investigates the characteristics and outcomes of organic and non-organic FTT among outpatients referred to the KAMC pediatric clinic. Notably, the study found a higher prevalence of non-organic FTT (54.15%) compared to organic FTT (45.84%). Among the 349 participants, 160 were classified as having organic causes, primarily related to gastrointestinal issues (27.2%) and endocrine problems (20.4%), with IUGR being the least common (7.4%). In the non-organic group, inadequate intake emerged as the dominant factor, followed by behavioral causes. 

Another study conducted between 2008 and 2013 in South Korea examined the clinical characteristics and outcomes of infants and toddlers diagnosed with FTT. In this study, 65% of the FTT cases were not due to non-organic FTT (NOFTT), while 35% were due to organic FTT [6]. Similar to our data on organic causes, gastroenterological disorders accounted for the majority of organic causes (11.4%). However, there was a difference in the proportion of patients with endocrinological and genetic disorders, which may be attributed to the different number of patients studied, as the South Korean study examined only 123 patients.

In a study conducted by another researcher, 70(63.6%) participants out of the total 110 participants were male [14]. This could be attributed to a higher number (53.9%) of male participants in the study population. In addition, 81 (89%) patients were found to have NOFTT, 10 had organic FTT, and 19 had insufficient information [14]. Compared to this study, NOFTT accounted for 189 (54.15%) of the participants, while 160(45.84%) were part of the organic group. A similar result was found in the study by Daniel et al., which showed that 51.5% of participants were male. This study also showed that 55.6% of the patients were between the ages of 6-18 months, 38.1% were between the ages of 19-30 months, and 2.1% were older than 42 months [15]. Compared to this study, male patients accounted for 53.9% of the participants while the mean age at referral was 5.2 years with the organic group presenting earlier at 4.7 years. compared to the non-organic group at a mean age of 5.6 years.

This study provides insights into the significant differences between organic and non-organic FTT outcomes. We found that the resolution of the non-organic group was significantly higher than that of the organic group. This shows that non-organic causes are easier to resolve before debilitating irreversible effects occur, unlike organic causes. Another major finding was the percentages of height and weight gain, which were the highest among the organic groups, particularly in children with IUGR.

One of the limitations of the study included investigating the duration required for resolution in both groups was beyond the scope of this study. Another limitation was that there is very little research in the field of FTT nationally, making it difficult to find papers to compare our results. Despite these limitations, our study was nonetheless able to answer our research questions and could identify significant differences between the organic and non-organic causes of FTT.

## Conclusions

This was a single-center study, showing that non-organic causes are more common and have better resolution outcomes. We recommend further multi-centered, nationally based research to compare the time windows to complete resolution of the causes of FTT. Further studies are needed on non-organic causes. Education for parents and children regarding proper nutrition is vital, as the majority of cases of FTT are attributed to non-organic factors, with insufficient intake being identified as the most common causative factor.
